# Supplementary material for: Entropy favors heterogeneous structures of networks near the rigidity threshold
Source: Nat Commun. 2018 Apr 10;9:1359. doi: 10.1038/s41467-018-03859-9 (PMC5893606; doi:10.1038/s41467-018-03859-9)
Supplement: Supplementary file 1 — Supplementary Information [file 41467_2018_3859_MOESM1_ESM.pdf]

Supplementary Information: Entropy favors heterogeneous structures of networks near the rigidity threshold

Le Yan*
Kavli Institute for Theoretical Physics, University of California, Santa Barbara, CA 93106, USA
(Dated: March 28, 2018)

SUPPLEMENTARY FIGURES

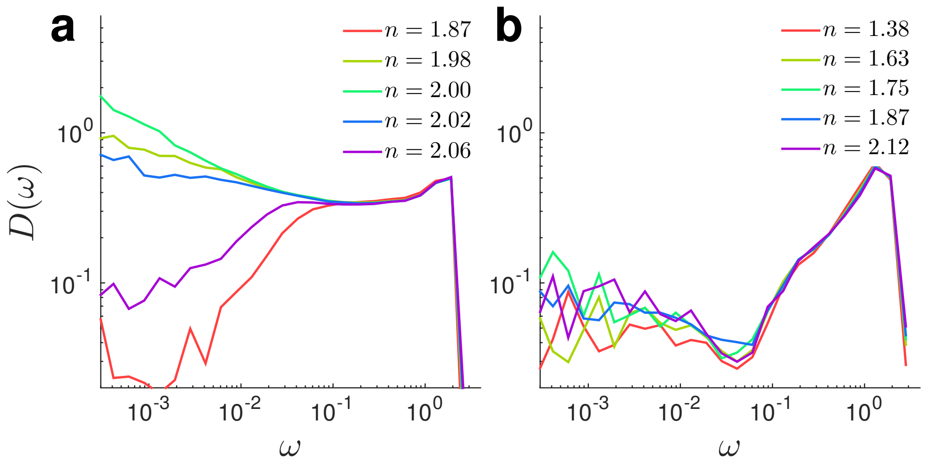

Supplementary Figure 1. Density of vibrational states in different scenarios. $D(\omega)$ for (a) self-organized homogeneous networks and (b) phase separated networks $\alpha = 0.0003$ with various number of constraints below and above the rigidity transition, $N = 576$. Vibrational frequencies are computed without including the weak forces.

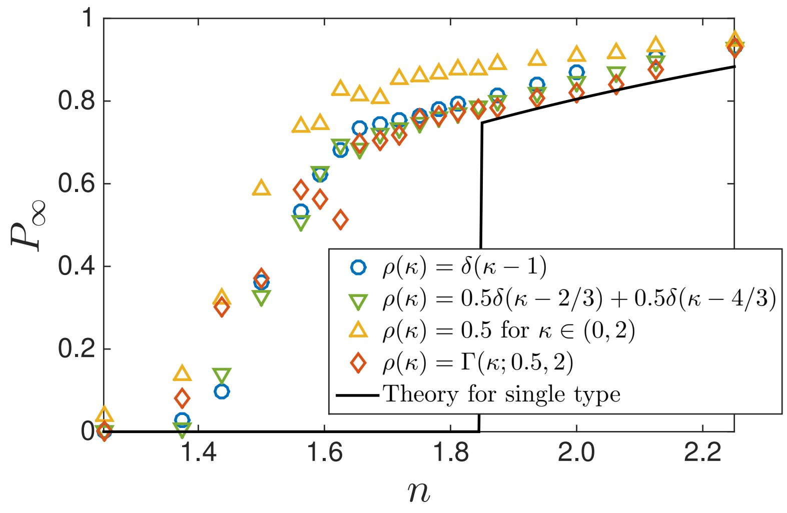

Supplementary Figure 2. Rigidity transition for different distributions of interactions. Probability of the bond in the rigidity percolating cluster P_∞ versus the average number of constraints n for $N = 256$. The stiffnesses of the constraints k are drawn from the distributions $\rho(\kappa)$, including single type (blue circles), two types (green triangles), uniform distribution (yellow triangles), and Gamma distribution (red diamonds).

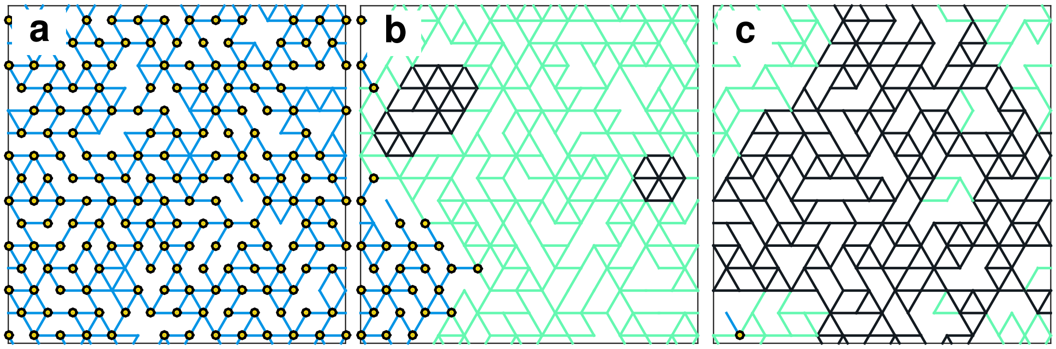

Supplementary Figure 3. Rigidity transition scenario in the nonlinear limit. Typical network structures (a) below, (b) at, and (c) above the Maxwell point n_c obtained with vibrational entropy in the nonlinear limit Supp. Eq.(31).

SUPPLEMENTARY NOTES

Supplementary Note 1. Linear approximation

Consider a network of N nodes connected by N_c springs. If an infinitesimal displacement field $|\delta\mathbf{R}\rangle$ is imposed on the nodes, the change of length of the springs can be written as a vector $|\delta r\rangle$ of dimension N_c. For small displacements, this relation is approximately linear: $|\delta r\rangle = \mathcal{S}|\delta\mathbf{R}\rangle$, where $\mathcal{S}$ is a $N_c \times Nd$ matrix. To simplify the notation, we write $\mathcal{S}$ as a $N_c \times N$ matrix of components of dimensions d, which gives $\mathcal{S}_{\gamma,i} \equiv \partial r_\gamma/\partial\mathbf{R}_i = \delta_{\gamma,i}\mathbf{n}_\gamma$, where $\delta_{\gamma,i}$ is non-zero only if the spring γ connects to the particle i, and $\mathbf{n}_\gamma$ is the unit vector in the direction of the spring γ, pointing toward the node i. Using the bra-ket notation, we can rewrite $\mathcal{S} = \sum_{\langle ij\rangle\equiv\gamma} |\gamma\rangle\mathbf{n}_\gamma(\langle i| - \langle j|)$, where the sum is over all the springs of the network. Note that the transpose $\mathcal{S}^t$ of $\mathcal{S}$ relates the set of contact forces $|f\rangle$ to the set $|\mathbf{F}\rangle$ of unbalanced forces on the nodes: $|\mathbf{F}\rangle = \mathcal{S}^t|f\rangle$, which simply follows from the fact that $\mathbf{F}_i = \sum_\gamma \delta_{\gamma,i} f_\gamma \mathbf{n}_\gamma = \sum_\gamma f_\gamma \mathcal{S}_{\gamma,i}$ [1].

Dynamic matrix. The dynamic matrix $\mathcal{M}$ is a linear operator connecting external forces to the displacements: $\mathcal{M}|\delta\mathbf{R}\rangle = |\mathbf{F}\rangle$. Introducing the $N_c \times N_c$ diagonal matrix $\mathcal{K}$, whose components are the spring stiffnesses $\mathcal{K}_{\gamma\gamma} = k_\gamma$, we have for harmonic springs $|f\rangle = \mathcal{K}|\delta r\rangle$. Applying $\mathcal{S}^t$ on each side of this equation, we get $|\mathbf{F}\rangle = \mathcal{S}^t|f\rangle = \mathcal{S}^t\mathcal{K}\mathcal{S}|\delta\mathbf{R}\rangle$, which thus implies [1]:

$$\mathcal{M} = \mathcal{S}^t\mathcal{K}\mathcal{S}. \tag{1}$$

Note that in our model the diagonal matrix $\mathcal{K}$ contains two types of coefficients k_w and k, corresponding to the stiffnesses of weak springs and strong springs determining the configurations of networks. Then the dynamic matrix can be written as $\mathcal{M} = k(\mathcal{S}_s^t\mathcal{S}_s + \frac{k_w}{k}\mathcal{S}_w^t\mathcal{S}_w)$, where $\mathcal{S}_w^t$ is the projection of the operator $\mathcal{S}^t$ on the subspace of weak springs. In the mean-field limit of weak interactions, number of weak neighbors $z_w \to \infty$ while keeping $\alpha \equiv z_w k_w/(kd)$ constant, the weak springs lead to an effective interaction between each node and the center of mass of the system [2], so that,

$$\mathcal{M} \approx k\left(\mathcal{S}_s^t\mathcal{S}_s + \alpha\mathcal{I}\right), \tag{2}$$

where $\mathcal{I}$ is a $dN \times dN$ identity matrix.

Therefore, the vibrational modes of the strong network $|\delta\mathbf{R}_\omega\rangle$,

$$\mathcal{M}_s|\delta\mathbf{R}_\omega\rangle = k\omega^2|\delta\mathbf{R}_\omega\rangle, \tag{3}$$

where $\mathcal{M}_s = \mathcal{S}_s^t\mathcal{S}_s$, are approximately the eigen vibrations of $\mathcal{M}$,

$$\mathcal{M}|\delta\mathbf{R}_\omega\rangle = \mathcal{M}_s|\delta\mathbf{R}_\omega\rangle + k\alpha|\delta\mathbf{R}_\omega\rangle = k(\omega^2 + \alpha)|\delta\mathbf{R}_\omega\rangle \tag{4}$$

with the eigenvalues lifted up by α.

Stress energy. The mismatches of link lengths to the spring rest lengths $|y\rangle$ generate an unbalanced force field $|\mathbf{F}\rangle = \mathcal{S}^t\mathcal{K}|y\rangle$ on the nodes, leading to a displacement $|\delta\mathbf{R}\rangle = \mathcal{M}^{-1}\mathcal{S}^t\mathcal{K}|y\rangle$. The elastic energy $H = \frac{1}{2}\langle y-\delta r|\mathcal{K}|y-\delta r\rangle$ is minimal for this displacement and the corresponding energy H_0 is:

$$H_0(|y\rangle) = \frac{1}{2}\langle y|\mathcal{K} - \mathcal{K}\mathcal{S}\mathcal{M}^{-1}\mathcal{S}^t\mathcal{K}|y\rangle. \tag{5}$$

In our model, $y_\gamma = 0$ for weak springs and $y_\gamma = \epsilon_\gamma$ is a Gaussian random variable for strong springs. Introducing $\mathcal{S}^t_{\mathrm{s}}$, the operator $\mathcal{S}^t$ on the subspace of strong springs of dimension N_{s}, we have $k\mathcal{S}^t_{\mathrm{s}}|\epsilon\rangle \equiv \mathcal{S}^t\mathcal{K}|y\rangle$ and Supp. Eq.(5) becomes

$$H_0(|\epsilon\rangle) = \frac{k}{2}\langle\epsilon|\mathcal{I} - k\mathcal{S}_{\mathrm{s}}\mathcal{M}^{-1}\mathcal{S}^t_{\mathrm{s}}|\epsilon\rangle. \tag{6}$$

Supplementary Note 2. Entropy gain λ

In two phase separation, the vibrational entropy reads

$$\frac{S_{\mathrm{vib}}}{N} = -V_{\mathrm{f}}(n_{\mathrm{c}} - n_{\mathrm{f}})\ln\omega_0 - n_{\mathrm{f}}V_{\mathrm{f}}\int \mathrm{d}\omega D_{\mathrm{f}}(\omega)\ln\omega - dV_{\mathrm{r}}\int \mathrm{d}\omega D_{\mathrm{r}}(\omega)\ln\omega. \tag{7}$$

ω_0 is the vibrational frequency of floppy modes, $\omega_0 = \sqrt{\alpha} > 0$ thanks to the weak interactions. In a weak field $\alpha \ll 1$, the other mode frequency is leveled up approximately as $\omega' = \sqrt{\omega^2 + \alpha}$. Density of non-floppy modes $D(\omega) \equiv \sum_{\omega'>\omega_0}\delta(\omega'-\omega)/\sum_{\omega'>\omega_0}$, $D_{\mathrm{f}}(\omega)$ and $D_{\mathrm{r}}(\omega)$ are densities of floppy and rigid phases accordingly.

The densities of states of entropy-favored networks are shown together with ones of homogeneous networks in Supp. Fig. 1. As predicted by the mean-field theory, the density of homogeneous networks is cutoff on the low frequency end at the boson peak $\omega^* \sim |n - n_{\mathrm{c}}|$, which is singular at the rigidity transition. For heterogeneous networks, the density of states presents no such singularity at the transition n^*. Like very stressed networks and very floppy ones, the density is blocked in high frequency modes cutoff at $\omega \sim 0.1$. An odd feature is the appearance of low frequency modes, shown as a flat distribution with quite low density. We speculate the feature is related to a tendency of small clusters organizing into one dimensional chains.

Though we have seen a significant change in the volume portion of the rigid phase and the floppy phase in the range of constraints number we prob, the densities of states for different numbers of constraints lay over on each other quite well, which implies that our approximation in Eq.(4) neglecting the difference between D_{f} and D_{r} is a good approximation.

Positive definiteness of λ. Consider creating a floppy mode in the floppy phase,

$$\Delta S_{\mathrm{vib}} = -\ln\omega_0 - Nd\int_{\omega_0}^{\omega_{\mathrm{D}}} \mathrm{d}\omega\Delta D(\omega)\ln\omega, \tag{8}$$

where the first term is the contribution from the floppy mode, while $\Delta D(\omega)$ in the second term includes the density shift of both D_{f} and D_{r} towards the Debye frequency ω_{D} when lowering the connectivity in the floppy phase and increasing n in the rigid phase [2, 3]. By definition, the total number of modes does not change, $Nd\int \mathrm{d}\omega\Delta D(\omega) = -1$. We decompose the variance of density of states in a special way

$$Nd\Delta D(\omega) = \rho_+(\omega) - \rho_-(\omega),$$

that both $\rho_+(\omega)$ and $\rho_-(\omega) \geq 0$ for $\forall\omega \in [\omega_0, \omega_{\mathrm{D}}]$, and $\int \mathrm{d}\omega\rho_-(\omega)\omega = \omega_0 + a\omega_{\mathrm{D}}$, where $a \equiv \int \mathrm{d}\omega\rho_+(\omega)$, so $\int \mathrm{d}\omega\rho_-(\omega) = 1 + a$. Then

$$\begin{aligned}\Delta S_{\mathrm{vib}} &= -\ln\omega_0 - \int \mathrm{d}\omega\rho_+(\omega)\ln\omega + \int \mathrm{d}\omega\rho_-(\omega)\ln(\omega) \geq -\ln\omega_0 - a\ln\omega_{\mathrm{D}} + \int_{\omega_0}^{\omega_{\mathrm{D}}} \mathrm{d}\omega\rho_-(\omega)\ln\omega \\ &\geq -\ln\omega_0 - a\ln\omega_{\mathrm{D}} + (1+a)\ln\omega_0 + \frac{\ln\omega_{\mathrm{D}} - \ln\omega_0}{\omega_{\mathrm{D}} - \omega_0}\left(\int \mathrm{d}\omega\rho_-(\omega)\omega - (1+a)\omega_0\right) = 0. \end{aligned}\tag{9}$$

In the second inequality, we have used the concaveness of $\ln\omega$, where the integral of $\ln\omega$ is larger than the integral of a linear function connecting the two end points. Defined as vibrational entropy gain per floppy mode,

$$\lambda \equiv \frac{\partial S_{\mathrm{vib}}}{\partial N_{\mathrm{f}}} \geq 0. \tag{10}$$

Self-stress prohibited. The entropy increases by creating isostatic region $\lambda' \geq 0$. By definition,

$$dN\lambda' = -\int_0^\infty \mathrm{d}\omega[\mathcal{N}_\mathrm{c}(\omega)-\mathcal{N}_\mathrm{f}(\omega)]\ln\omega+(n_\mathrm{c}-n_\mathrm{f})\int_0^\infty \mathrm{d}\omega\partial_{n_\mathrm{f}}\mathcal{N}_\mathrm{f}(\omega)\ln\omega = -\int_0^\infty \mathrm{d}\omega\int_{n_\mathrm{f}}^{n_\mathrm{c}}\mathrm{d}n\int_{n_\mathrm{f}}^{n}\mathrm{d}n'\partial_{n'}^2\mathcal{N}_{n'}(\omega)\ln\omega \quad (11)$$

where $\mathcal{N}_n(\omega) = nND(\omega)$ counts the number of vibrations ω for homogeneous network of constraint number n. As n increases by $\mathrm{d}n > 0$ for $n < n_\mathrm{c}$, about $N\mathrm{d}n$ vibrations emerge at $\omega \sim n_\mathrm{c} - n$ [3], $\mathcal{N}_{n+\mathrm{d}n}(\omega) - \mathcal{N}_n(\omega) \geq 0$ for $\forall\omega$. Equivalently, $\partial_n\mathcal{N}_n(\omega) \approx b\partial_\omega\mathcal{N}_n(\omega)$, with $b > 0$. Therefore,

$$dN\lambda' \approx -b^2\int_0^\infty \mathrm{d}\omega\int_{n_\mathrm{f}}^{n_\mathrm{c}}\mathrm{d}n\int_{n_\mathrm{f}}^{n}\mathrm{d}n'\mathcal{N}_{n'}(\omega)\partial_\omega^2\ln\omega \geq 0 \quad (12)$$

The inequality is again the result of concaveness of log function, $\partial_\omega^2\ln\omega < 0$.

Specifically, we consider an approximation to the density of states in random networks of constraint number n: a flat density $D_0(\omega) = 1/b$ cut off at $\omega^* = b(n_\mathrm{c} - n)$ and $\omega_\mathrm{D} = bn_\mathrm{c}$ [3], where $b \sim 1$.

$$\frac{S_\mathrm{vib}}{Nd} \approx -\frac{V_\mathrm{c}}{2\omega_\mathrm{D}}\int_0^{\omega_\mathrm{D}}\mathrm{d}\omega\ln(\omega^2+\alpha) - \frac{V_\mathrm{f}}{2\omega_\mathrm{D}}\int_{b(n_\mathrm{c}-n_\mathrm{f})}^{\omega_\mathrm{D}}\mathrm{d}\omega\ln(\omega^2+\alpha). \quad (13)$$

$$\lambda' = \frac{1}{2\omega_\mathrm{D}}\int_0^{b(n_\mathrm{c}-n_\mathrm{f})}\mathrm{d}\omega\ln\frac{b^2(n_\mathrm{c}-n_\mathrm{f})^2+\alpha}{\omega^2+\alpha} = \frac{1}{\omega_\mathrm{D}}\left[b(n_\mathrm{c}-n_\mathrm{f}) - \sqrt{\alpha}\arctan\frac{b(n_\mathrm{c}-n_\mathrm{f})}{\sqrt{\alpha}}\right] \geq 0. \quad (14)$$

$\lambda' \approx \frac{b^3}{3\alpha\omega_\mathrm{D}}(n_\mathrm{c}-n_\mathrm{f})^3$ for $n_\mathrm{c}-n_\mathrm{f} \lesssim \sqrt{\alpha}/b$ and $\lambda' \approx \frac{b}{\omega_\mathrm{D}}(n_\mathrm{c}-n_\mathrm{f})$ for $n_\mathrm{c}-n_\mathrm{f} \gtrsim \sqrt{\alpha}/b$.

Supplementary Note 3. Free energy at T

For simplicity, we consider the annealed free energy $F_\mathrm{ann} = -T\ln\overline{\mathcal{Z}}$. It is exact in the random energy model [4] above the ideal glass transition [5] and we find it to be a good approximation of F in network models [6]. The over-line implies an average over quenched disorder ϵ,

$$\overline{\mathcal{Z}} = \sum_\Gamma \overline{\exp[-F(\Gamma)/T]}^\epsilon. \quad (15)$$

Applying the linear approximation Supp. Eq.(6) and the Gaussian distribution $\rho(\epsilon_\gamma) = \frac{1}{\sqrt{2\pi\epsilon^2}}e^{-\epsilon_\gamma^2/2\epsilon^2}$ of frustration at bond γ, we have

$$\overline{\mathcal{Z}} = \sum_\Gamma \exp\left[-\frac{1}{2}\,\mathrm{tr}\ln\left(\mathcal{I}+\frac{\mathcal{G}(\Gamma)}{T}\right) + S_\mathrm{vib}(\Gamma)\right]. \quad (16)$$

where we have used $k\epsilon^2 = 1$ to scale the temperature. As shown in Supp. Eq.(21), when $\alpha = 0$, coupling matrix $\mathcal{G}$ acts as a projection operator onto the null space of structure matrix $\mathcal{S}_\mathrm{s}$. So

$$-\frac{F}{NT} = \frac{S_\mathrm{conf}}{N} + \frac{S_\mathrm{vib}}{N} - \frac{V_\mathrm{r}(n_\mathrm{r}-n_\mathrm{c})}{2}\ln\left(1+\frac{1}{T}\right), \quad (17)$$

for each self-stress direction created, free energy decreases by $\lambda_\mathrm{F} = \lambda - \frac{1}{2}\ln(1+\frac{1}{T})$.

Including the perturbation $\alpha > 0$ in the floppy region $n < n_\mathrm{c}$, the total free energy for isostatic-floppy separation [6] then follows

$$\frac{F}{NT} = \frac{V_\mathrm{c}n_\mathrm{c}}{2}\int \mathrm{d}\omega D_\mathrm{c}(\omega)\ln\left(1+\frac{\alpha}{\alpha+\omega^2}\frac{1}{T}\right) + \frac{V_\mathrm{f}n_\mathrm{f}}{2}\int \mathrm{d}\omega D_\mathrm{f}(\omega)\ln\left(1+\frac{\alpha}{\alpha+\omega^2}\frac{1}{T}\right) + \frac{n_\mathrm{c}-n}{2}\ln\left(1+\frac{1}{T}\right) - \frac{S_\mathrm{vib}}{N} - \frac{S_\mathrm{conf}}{N}. \quad (18)$$

So the free energy loss,

$$\lambda'_\mathrm{F} = -\frac{\partial F/NT}{d\partial V_\mathrm{c}} \approx \lambda' - \frac{1}{2\omega_\mathrm{D}}\int_0^{b(n_\mathrm{c}-n_\mathrm{f})}\mathrm{d}\omega\ln\frac{1+\frac{\alpha/T}{\alpha+\omega^2}}{1+\frac{\alpha/T}{\alpha+b^2(n_\mathrm{c}-n_\mathrm{f})^2}} = \frac{1}{\omega_\mathrm{D}}\left(b(n_\mathrm{c}-n_\mathrm{f}) - \sqrt{\alpha(1+\frac{1}{T})}\arctan\frac{b(n_\mathrm{c}-n_\mathrm{f})}{\sqrt{\alpha(1+1/T)}}\right), \quad (19)$$

becomes approximately linear in $n_\mathrm{c}-n_\mathrm{f}$ when $n_\mathrm{c}-n_\mathrm{f} \gtrsim \sqrt{\alpha}/b$, faster than the heterogeneous boundary $\Lambda \sim (n_\mathrm{c}-n_\mathrm{f})^2$ in Eq.(8).

Supplementary Note 4. Shear modulus of perturbed networks

We consider elastic model approximation $T_{\rm g} \sim G$ [7] for the glass transition temperature $T_{\rm g}$. Here, we derive the scaling relations of G, n and α from a perturbation theory. In the linear approximation Supp. Eq.(5), the elastic energy $\mathcal{H}_0$ is quadratic to any associated deformation $|y\rangle$. For a shear in x-y plane,

$$|y\rangle = \gamma \left| \frac{\Delta x \Delta y}{\Delta r} \right\rangle,$$

where γ is the shear strain, Δx, Δy and Δr are the projection onto x and y directions and the length of the corresponding springs.

Shear modulus of a configuration Γ,

$$G(\Gamma) = \frac{1}{V}\frac{\partial^2 \mathcal{H}_0(\Gamma)}{\partial \gamma^2} = \frac{1}{V}\left\langle \frac{\Delta x \Delta y}{\Delta r} \right| \mathcal{G} \left| \frac{\Delta x \Delta y}{\Delta r} \right\rangle \tag{20}$$

where $\mathcal{G} = \mathcal{K} - \mathcal{K}\mathcal{S}\mathcal{M}^{-1}\mathcal{S}^t\mathcal{K}$ depends on the configuration of the network. We can decompose the stiffness matrix $\mathcal{K}$ and the structure matrix $\mathcal{S}$ onto the strong and weak connections,

$$\mathcal{K} = \begin{pmatrix} k\mathcal{I}_{\rm s} & 0 \\ 0 & k_{\rm w}\mathcal{I}_{\rm w} \end{pmatrix}, \qquad \mathcal{S} = \begin{pmatrix} \mathcal{S}_{\rm s} \\ \mathcal{S}_{\rm w} \end{pmatrix}.$$

From the approximation of the dynamic matrix $\mathcal{M}$ in Supp. Eq.(2), we can decompose it as

$$\mathcal{M} = k\sum_\omega (\omega^2 + \alpha)|\delta \mathbf{R}_\omega\rangle\langle \delta \mathbf{R}_\omega|.$$

Similarly, we write $\mathcal{I}_{\rm s}$ and $\mathcal{S}_{\rm s}$ in the same basis and corresponding basis in connection space $|\delta r_\omega\rangle = \frac{1}{\omega}\mathcal{S}_{\rm s}|\delta \mathbf{R}_\omega\rangle$,

$$\mathcal{S}_{\rm s} = \sum_\omega \omega |\delta r_\omega\rangle\langle \delta \mathbf{R}_\omega|, \quad \mathcal{I}_{\rm s} = \sum_p |\psi_p\rangle\langle\psi_p| + \sum_\omega |\delta r_\omega\rangle\langle \delta r_\omega|$$

where $|\psi_p\rangle$ defines the null space of the structure $\mathcal{S}_{\rm s}$ that self-stresses live in. We then get,

$$G(\Gamma) = \frac{1}{V}\left(k\sum_p |X_p|^2 + k\sum_\omega \frac{\alpha}{\omega^2+\alpha}|X_\omega|^2 + N_{\rm w}k_{\rm w}a_{\rm w}^2 - 2k_{\rm w}\sum_\omega \frac{\omega}{\omega^2+\alpha} X_\omega X_\omega^{\rm w} + o(k_{\rm w}^2) \right), \tag{21}$$

where $X_p = \langle \psi_p | \frac{\Delta x \Delta y}{\Delta r}\rangle$, $X_\omega = \langle \delta r_\omega | \frac{\Delta x \Delta y}{\Delta r}\rangle$, $N_{\rm w}a_{\rm w}^2 = \sum_{\rm weak}\langle \frac{\Delta x \Delta y}{\Delta r} | \frac{\Delta x \Delta y}{\Delta r}\rangle$, and $X_\omega^{\rm w} = \langle \frac{\Delta x \Delta y}{\Delta r} | \mathcal{S}_{\rm w} | \delta \mathbf{R}_\omega\rangle$.

Finally, we average over the configurations. For the isotropic disordered networks we are dealing with, $\frac{\Delta x \Delta y}{\Delta r}$ should be a random variable distributed evenly around zero independent of the choices of basis. X_p, X_ω are thus sums of $N_{\rm s}$ random variables with zero mean. Central Limit Theorem thus gives,

$$G = \begin{cases} \rho n_{\rm c} k a^2 \left(\frac{n}{n_{\rm c}} - 1 + \int {\rm d}\omega D(\omega)\frac{\alpha}{\omega^2+\alpha} + \alpha\frac{a_{\rm w}^2}{2a^2} \right) & n > n_{\rm c} \\ \rho n_{\rm c} k a^2 \left(\frac{n}{n_{\rm c}} \int {\rm d}\omega D(\omega)\frac{\alpha}{\omega^2+\alpha} + \alpha\frac{a_{\rm w}^2}{2a^2} \right) & n < n_{\rm c} \end{cases} \tag{22}$$

where $\rho = N/V$, a^2 is the variance of X_p and X_ω, and $D(\omega)$ is normalized density of vibrational states. For perturbative $\alpha \ll 1$, the shear modulus $G \propto n - n_{\rm c}$ for $n > n_{\rm c}$, and $G \sim \alpha$ when $n < n_{\rm c}$.

Supplementary Note 5. General interactions

We generalize our results to elastic networks of dispersed interactions, still assuming the separation of energy scales. Each pair of neighboring particles either interact through a bond of strength κ from some distribution $\rho(\kappa)$ or do not interact,

$$P(\kappa) = (1-p)\delta(\kappa) + p\rho(\kappa). \tag{23}$$

where δ is Dirac delta function. When phases separate, we have a rigid phase of volume V_{r} with connections characterized by a distribution $P_{\mathrm{r}}(\kappa)$ and a floppy phase of volume V_{f} and distribution $P_{\mathrm{f}}(\kappa)$. They are constrained by

$$V_{\mathrm{f}} + V_{\mathrm{r}} = 1 \tag{24a}$$
$$V_{\mathrm{f}} P_{\mathrm{f}}(\kappa) + V_{\mathrm{r}} P_{\mathrm{r}}(\kappa) = P(\kappa) \tag{24b}$$
$$\int \mathrm{d}\kappa P_{\mathrm{f}}(\kappa) = \int \mathrm{d}\kappa P_{\mathrm{r}}(\kappa) = 1. \tag{24c}$$

The configuration entropy of the layout is,

$$\frac{S_{\mathrm{conf}}}{N} = -n_{\mathrm{m}} \left(V_{\mathrm{f}} \int \mathrm{d}\kappa P_{\mathrm{f}}(\kappa) \ln P_{\mathrm{f}}(\kappa) + V_{\mathrm{r}} \int \mathrm{d}\kappa P_{\mathrm{r}}(\kappa) \ln P_{\mathrm{r}}(\kappa) \right) \tag{25}$$

Without loss of generality, we consider the vibrational entropy following

$$\frac{S_{\mathrm{vib}}}{N} = V_{\mathrm{f}} \Lambda \int \mathrm{d}k \eta_{\mathrm{f}}(\kappa) P_{\mathrm{f}}(\kappa) + V_{\mathrm{r}} \Lambda \int \mathrm{d}\kappa \eta_{\mathrm{r}}(\kappa) P_{\mathrm{r}}(\kappa) = V_{\mathrm{f}} \Lambda \int \mathrm{d}\kappa \eta(\kappa) P_{\mathrm{f}}(\kappa) + s_0, \tag{26}$$

where $\eta(\kappa) = \eta_{\mathrm{f}}(\kappa) - \eta_{\mathrm{r}}(\kappa)$ and $s_0 = \Lambda \int \mathrm{d}\kappa \eta_{\mathrm{r}}(\kappa) P(\kappa)$. This linear assumption, however, may just be approximately true, especially when the segregation of weak interactions appear, which is accompanied with a diverging density of soft vibrations [8] and contributes nonlinearly. We define a marginal network $P_{\mathrm{c}}(\kappa)$ by where the vibrational entropy equals to zero, $\int \mathrm{d}\kappa \eta(\kappa) P_{\mathrm{c}}(\kappa) = 0$.

All together, the total entropy $S_{\mathrm{vib}} + S_{\mathrm{conf}}$ is optimized by,

$$\Lambda \eta(\kappa) = \ln \frac{P_{\mathrm{f}}(\kappa)}{P_{\mathrm{r}}(\kappa)}. \tag{27}$$

Multiplying both side of Supp. Eq.(27) with $P_{\mathrm{c}}(\kappa)$ and integrate over κ, we find a balance condition,

$$D(P_{\mathrm{c}}|P_{\mathrm{f}}) = \int \mathrm{d}\kappa P_{\mathrm{c}}(\kappa) \ln \frac{P_{\mathrm{c}}(\kappa)}{P_{\mathrm{f}}(\kappa)} = D(P_{\mathrm{c}}|P_{\mathrm{r}}), \tag{28}$$

the relative entropies [5] to the critical distribution of the distributions in the rigid and floppy phases are equal. Similarly, we have,

$$\Lambda \int \mathrm{d}\kappa \eta(\kappa) P_{\mathrm{f}}(\kappa) = \int \mathrm{d}\kappa P_{\mathrm{f}}(\kappa) \ln \frac{P_{\mathrm{f}}(\kappa)}{P_{\mathrm{r}}(\kappa)} = D(P_{\mathrm{f}}|Pr), \tag{29}$$

entropic gain per unit volume in floppy phase compensates the relative entropy from the rigid phase to the floppy one.

The general results apply to specific cases. In the network of a single type strong interaction, we have $P(\kappa) = \frac{n_{\mathrm{m}}-n}{n_{\mathrm{m}}}\delta(\kappa) + \frac{n}{n_{\mathrm{m}}}\delta(\kappa - k)$ and $\eta(\kappa) = \frac{n_{\mathrm{c}}}{n_{\mathrm{m}}}\delta(\kappa) - \frac{n_{\mathrm{m}}-n_{\mathrm{c}}}{n_{\mathrm{m}}}\delta(\kappa - k)$. In the network of compounds $A_x B_{1-x}$, κ labels different chemical elements, $P(\kappa) = \rho^A \delta_{\kappa,A} + \rho^B \delta_{\kappa,B}$ and $\eta(\kappa) = (n_{\mathrm{c}} - n^B)\delta_{\kappa,B} + (n_{\mathrm{c}} - n^A)\delta_{\kappa,A}$, where δ is Kronecker delta symbol.

Numerical evidence of separation. We confirm numerically the robustness of our prediction on phase separation independent of our choice of single type of strong interactions. We have considered the bi-disperse, uniformly distributed, and Gamma distributed interaction strengths. As shown in Supp. Fig. 2, independent of the choice of the distributions, the rigidity consistently percolates below the Maxwell point $n_{\mathrm{c}} = 2$, because of the existence of the highly-connected rigid phase resulted from the phase separation.

SUPPLEMENTARY DISCUSSION

In the main text, we have focused on the thermal vibrations in the linear range in Eq.(2), valid in the low temperature limit. In order to see when the conclusions are valid and how entropy directs the network organization in the high temperature limit, we consider the nonlinear responses acting as a cutoff, than which the range of the linear vibration

T/ω^2 can not be larger. It's reasonable to assume that the nonlinear response starts to effect when the relative displacement of atoms is larger than the Lindemann's criterion [9], about 0.15 time of the typical atom distance.

$$S_{\rm vib}(\Gamma) = \sum_{\omega} \min[-\ln\omega + \frac{1}{2}\ln T, -\frac{1}{d}\ln P(\omega) + c] \tag{30}$$

where $P(\omega) = \sum_i \delta\mathbf{R}_i(\omega)^4$ is the participation ratio of the corresponding eigenmode $\delta\mathbf{R}_i(\omega)$, which estimates the number of atoms involved in given mode. So $P(\omega)^{-1/d}$ gives the relative displacement of two neighbors in the unit of Lindemann's distance. c is the constant determined by the range of nonlinear response.

The linear limit Eq.(2) breaks down at the high temperate $\ln T \gtrsim 2c$, where vibrational phase space is cutoff by the nonlinear response in each degrees of freedom,

$$S_{\rm vib} = -\frac{1}{d}\sum_{\omega} \ln P(\omega). \tag{31}$$

The typical structures maximizing Supp. Eq.(31) are shown in Supp. Fig. 3. In contrast to the heterogeneous effect of vibrational entropy discussed in the main text, it improves the homogeneity of the network structures, and the rigidity of the resulted networks again converges to the scenario discussed in mean-field theory with a sharp jump P_∞ at $n_{\rm c}$.

SUPPLEMENTARY REFERENCES

* lyan@kitp.ucsb.edu

[1] C.R. Calladine. Buckminster fuller's "tensegrity" structures and Clerk Maxwell's rules for the construction of stiff frames. *Int. J. Solids Struc.*, 14:161–172, (1978).
[2] M. Wyart, H. Liang, A. Kabla, and L. Mahadevan. Elasticity of floppy and stiff random networks. *Phys. Rev. Lett.*, 101:215501, (2008).
[3] G. Düring, E. Lerner, and M. Wyart. Phonon gap and localization lengths in floppy materials. *Soft Matter*, 9:146–154, (2013).
[4] B. Derrida. Random-energy model: An exactly solvable model of disordered systems. *Phys. Rev. B*, 24:2613–2626, (1981).
[5] A. Montanari and M. Mézard. *Information, Physics and Computation.* Oxford University press, (2009).
[6] L. Yan, G. Düring, and M. Wyart. Why glass elasticity affects the thermodynamics and fragility of supercooled liquids. *Proc. Natl. Acad. Sci. USA*, 110:6307–6312, (2013).
[7] J.C. Dyre. Colloquium: The glass transition and elastic models of glass-forming liquids. *Rev. Mod. Phys.*, 78:953–972, (2006).
[8] E. DeGiuli, E. Lerner, C. Brito, and M. Wyart. Force distribution affects vibrational properties in hard-sphere glasses. *Proc. Natl. Acad. Sci. USA*, 111:17054–17059, (2014).
[9] F.A. Lindemann. The calculation of molecular vibration frequencies. *Physik. Z.*, 11:609, (1910).
